# SPEECHLESS and MUTE Mediate Feedback Regulation of Signal Transduction during Stomatal Development

**DOI:** 10.3390/plants10030432

**Published:** 2021-02-24

**Authors:** Abdul Wakeel, Lin Wang, Ming Xu

**Affiliations:** 1 Henan Key Laboratory of Earth System Observation and Modeling, Henan University, Kaifeng 475004, China; awzju@yahoo.com; 2 College of Environment and Planning, Henan University, Kaifeng 475004, China; 3 Miami College, Henan University, Kaifeng 475004, China

**Keywords:** SPEECHLESS, MUTE, FAMA, bHLH, stomatal lineage, stomatal development

## Abstract

Stomatal density, spacing, and patterning greatly influence the efficiency of gas exchange, photosynthesis, and water economy. They are regulated by a complex of extracellular and intracellular factors through the signaling pathways. After binding the extracellular epidermal patterning factor 1 (EPF1) and 2 (EPF2) as ligands, the receptor-ligand complexes activate by phosphorylation through the MAP-kinase cascades, regulating basic helix-loop-helix (bHLH) transcription factors SPEECHLESS (SPCH), MUTE, and FAMA. In this review, we summarize the molecular mechanisms and signal transduction pathways running within the transition of the protodermal cell into a pair of guard cells with a space (aperture) between them, called a stoma, comprising asymmetric and symmetric cell divisions and draw several functional models. The feedback mechanisms involving the bHLH factors SPCH and MUTE are not fully recognized yet. We show the feedback mechanisms driven by SPCH and MUTE in the regulation of EPF2 and the ERECTA family. Intersections of the molecular mechanisms for fate determination of stomatal lineage cells with the role of core cell cycle-related genes and stabilization of SPCH and MUTE are also reported.

## 1. Introduction

*Arabidopsis thaliana* (*Arabidopsis*) protodermal leaf cells differentiate into three main cell types: unicellular leaf hairs (trichomes), pavement cells, and pairs of stomatal GCs that facilitate transpiration and exchange of gases between plant and atmosphere [1,2,3]. Stomata development from leaf protodermal cells follows the single-cell spacing rule to ensure the separation of stomata with at least a single pavement cell [4,5]. Stomata formation comprises a series of steps called stomatal lineage [6]. Stomatal lineage cells may undergo normal stomatal development by following single-cell space rules, exit the lineage, be arrested at any developmental stage, or undergo apoptosis by activating programmed cell death [3,7,8]. The cell cycle is checked and regulated by cell cycle checkpoints, which are present between each phase of the cell cycle, and decide the possible developmental fate of a cell [7].

**Figure 1:**
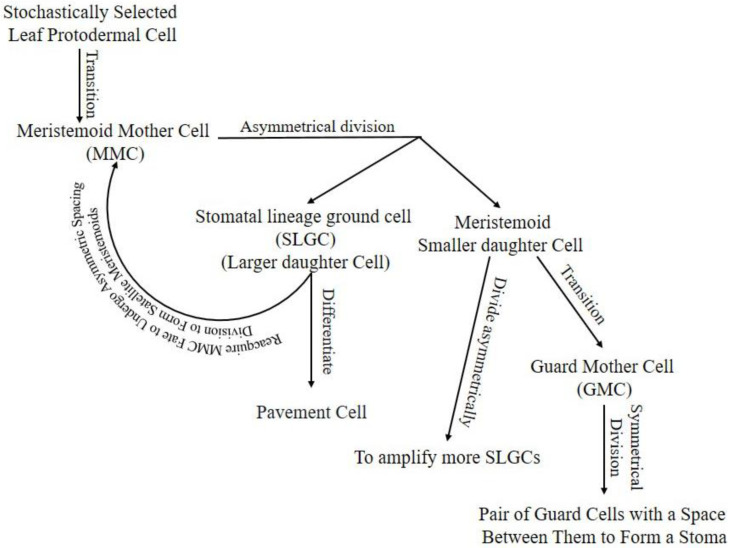
Stomatal lineage, starting from protodermal cell to pair of GCs enclosing stomata. Arrow lines indicate the progression of cells in the lineage. Hypothetically, the stomatal lineage ground cell (SLGC) will differentiate into a pavement cell if its neighboring cell is a meristemoid from another cell division. If the SLGC neighbor cell is an SLGC from another cell division, it will undergo a spacing division. Hypothetically, the meristemoid will progress into guard mother cells (GMC) if it is surrounded by SLGCs or pavement cells; otherwise, it will continue amplifying to attain enough SLGCs for a single-celled space rule. Cells may exit lineage in any stage due to unfavorable intrinsic or extrinsic conditions.

Stomatal lineage starts with stochastically selected leaf protodermal cell transition into the meristemoid mother cell (MMC). The MMC divides asymmetrically into a large daughter cell called a stomatal lineage ground cell (SLGC) and a meristemoid small daughter cell [5]. The SLGC in the daughter cells pair can differentiate into a pavement cell or reacquire MMC fate to undergo asymmetric spacing division to form satellite meristemoids [9]. Meristemoids can divide asymmetrically to amplify more SLGCs, transit into guard mother cells (GMC), or, rarely, exit the lineage [3]. The GMC will divide symmetrically into a pair of GCs with space between them to form a stoma, or its development can be arrested (Figure 1).

## 2. Role of SPEECHLESS (SPCH), MUTE, and FAMA in Stomata Development

**Figure 2:**
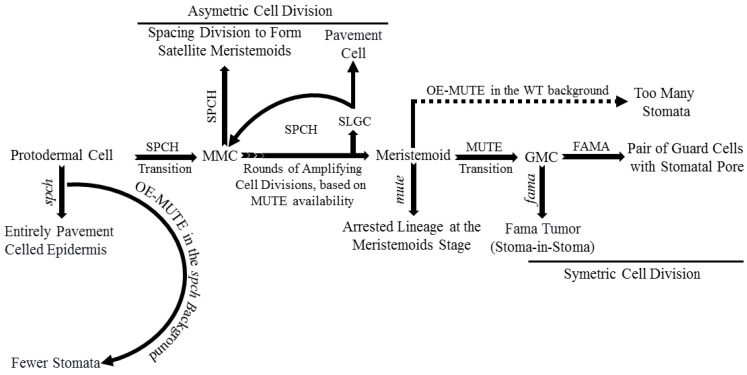
Role of basic helix-loop-helix (bHLH) transcription factors: SPCH, MUTE, and FAMA in stomatal development. SPCH regulates protodermal cells’ transition to meristemoid mother cell (MMC), rounds of amplifying cell division, and spacing division to form satellite meristemoids. The defective SPCH mutant exhibits pavement-celled epidermis entirely, whereas overexpression of MUTE in the SPCH background exhibits epidermis with the fewer-stomata phenotype. MUTE facilitates the meristemoid transition to GMC. The defective MUTE mutant arrests cell lineage at the meristemoid stage, whereas the overexpression of MUTE in the wild-type (WT) background shows a phenotype of too many stomata. FAMA plays a vital role in GMC’s symmetric division into a pair of GCs with a stomatal opening between them. The defective FAMA mutant presents a stomata-in-stomata or FAMA-tumor phenotype.

Basic helix-loop-helix (bHLH) transcription factors (TFs) such as SPEECHLESS (SPCH), MUTE, and FAMA are critical for stomatal development. Mutations in any one of these TFs result in improperly developed stomata [6,10,11,12]. SPCH regulates the transition of protodermal cells into MMCs and asymmetric cell division subsequently. SPCH-defective mutants were unable to initiate stomatal lineage, with an entirely pavement-celled epidermis phenotype. The different fate decisions of the meristemoid cells (amplifying division, spacing division, or progression down the lineage) depends on the activity and available level of SPCH (Figure 2) [12,13]. MUTE regulates meristemoids’ transition into GMCs, confirmed by arrested lineage at the meristemoid stage in a MUTE-defective mutant. Overexpression of MUTE in the *spch* background partially rescued spch-phenotype, and in the wild-type, it exhibited a phenotype, an epidermis densely populated with stomata (Figure 2) [10,14]. These results demonstrate that MUTE has no role in SPCH-regulated asymmetric amplification and spacing cell division [14].

FAMA regulates symmetric cell division of each GMC into a pair of GCs with a space between them to form stomata and terminates lineage cell meristematic activity [11,15,16]. Defective FAMA exhibited uncontrolled symmetric cell division of GMCs with a FAMA-tumor phenotype. FAMA-RETROBLASTOMA-RELATED (RBR) interaction facilitates POLYCOMB REPRESSOR COMPLEX-mediated chromatin methylation to switch off SPCH and MUTE expression. Incorrect FAMA expression resulted in a stoma-in-stoma phenotype, where GCs were pushed into lineage by active SPCH (Figure 2) [11,15,16,17]. Furthermore, FAMA-activated genes such as POTASSIUM CHANNEL IN *ARABIDOPSIS THALIANA* 1 are required for GCs functioning and control of stomatal aperture [17].

## 3. Ligand, Receptor, MPK Cascade, and Associated Pathways in Stomatal Development

**Figure 3:**
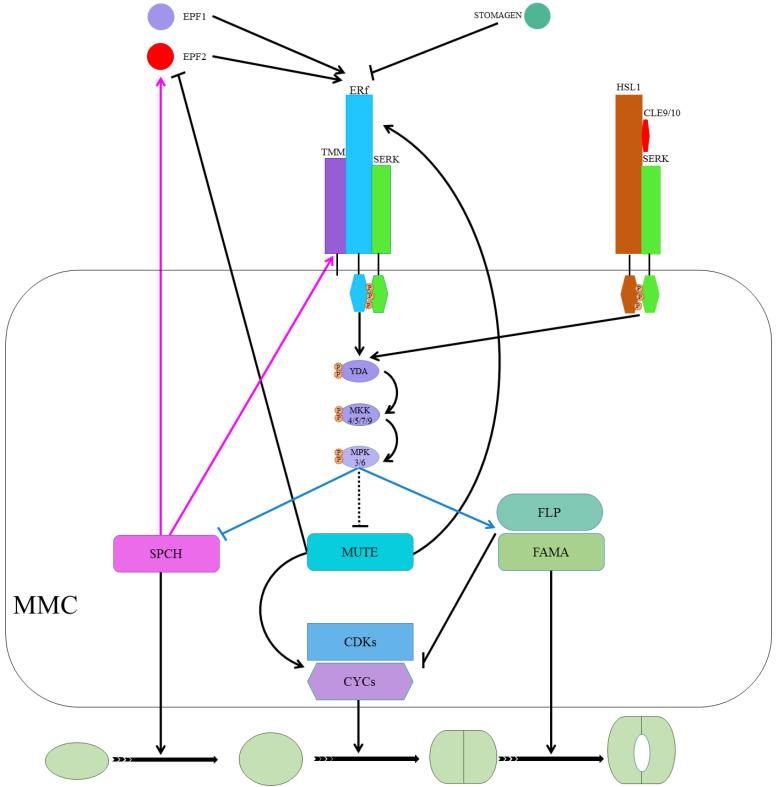
The ligand-receptor interactions are regulating SPCH, MUTE, and FAMA. Ligand (EPF1 and EPF2) binds to the receptor complex (ERf/TMM/SERK). It activates the MPK cascade (YDA-MKK-MPK) that suppresses SPCH, probably suppresses MUTE, and promotes FAMA for respective action during stomatal development. SPCH induces the expression of EPF2 and TMM in a negative feedback mechanism. MUTE induces ERLI and suppresses EPF2. ERL1 regulates meristemoid transition into GMC upon perceiving EPF1. MUTE upregulates CYC and CDK symmetric cell divisions of GMCs. MUTE locks in the cells and upregulates FLP and FAMA in the differentiation program that suppresses the cell cycle’s regulators to control single symmetric cell division. On the right, CLE9/10 is perceived by HSL1 and SERK complex, and signals from this activated complex result in SPCH phosphorylation and destabilization.

Peptide signaling, a well-known stomatal development regulator, is underpinned by EPIDERMAL PATTERNING FACTORS 1 (EPF1), EPF2, and EPIDERMAL PATTERNING FACTOR LIKE 9 (EPFL9, denoted as STOMAGEN, STOM) [18,19,20,21,22]. EPF1/2, as negative stomatal development regulators, bind/activate leucine-rich repeat receptor kinases (LRR-RKs). At the same time, STOM competes to bind the LRR-RKs with the EPF1/2 to positively regulate stomatal development [23,24,25]. EPF2 mainly regulates SPCH and the subsequent behavior of meristemoids, whereas EPF1 regulates the one-cell-spacing rule and participates in GMC’s autocrine regulation and inhibition of SLGCs from re-entering the stomatal lineage [26,27]. The ERECTA (ER) family, comprising ER, ERECTA-LIKE 1 (ERL1), and ERL2, are extensively studied LRR-RKs [28]. ERLI and ER play a role in the GMC development stage and facilitate the cell-specific MUTE activity, whereas ERL2 regulates SPCH, mainly as shown in Figure 3 [26,29]. The *erl1* and *erl2* single mutant and *erl1 erl2* double mutant showed reduced SLGC, a less severe phenotype as compared to the *er* single mutant. The less severe phenotype in ERL mutants than ER mutants demonstrates the ER dominance during stomatal development [30]. Increased stomatal density and index in the *er erl1* double mutant and elevated SLGCs in *er* mutant indicate that they are collectively regulating MUTE in the GMC stage. While slightly increased phenotype in the *er erl2* double mutants compared to *er*, the single mutant suggests that *erl2* is one of the main SPCH regulators during stomatal development [30].

TOO MANY MOUTHS (TMM) binds to the ER family (ER and ERL1) to form active extracellular complexes to perceive EPF1 and EPF2 for regulating correct stomatal development [31,32]. TMM, which is mainly expressed in young GCs and stomatal precursor cells, is part of the autoregulatory mechanism and a direct target of SPCH transcription regulators [12,33]. SOMATIC EMBRYOGENESIS RECEPTOR KINASES (SERKs), an irrelative LRR-RK family, have been found required for coupling extracellular TMM/ER complexes to perceive peptide signaling for downstream intracellular signaling, as shown in Figure 3 [34]. There are five homologs (SERK1-5) in *Arabidopsis*, and single mutants of these genes did not alter stomatal development. However, *serk1-1/serk2-1/SERK3-4* triple and *serk1-1/serk2-1/SERK3-4/SERK3-5* quadruple mutants displayed clustered stomata phenotypes, and the phenotype for the latter was similar to *er erl1 erl2* triple mutants [35].

Upon completion of the ER/SERK/TMM/EPF complex, the ER/SERK cytosolic kinase domain is phosphorylated to target mitogen-activated protein kinase kinase kinase (MPKKK), which is also known as YODA (YDA), a class MPK cascade [2,36,37,38]. YDA, in turn, phosphorylates mitogen-activated protein kinase kinases (MPKKs) that phosphorylate mitogen-activated protein kinase (MPKs) that directly phosphorylate the MPK cascade’s final target [3,37,38]. Only four (MPKK4/5/7/9) and three (MPK3/4/6), out of 20 in *Arabidopsis*, are involved in stomatal development [37,38,39]. MPK3/6 interacted with SPCH and MPK4 with MUTE, but no in vivo or phenotypic evidence for the latter has been reported previously [36,37,40]. The MPK3/6 was unable to interact with truncated SPCH without MAPK target domain (MPKTD) and exhibited clustered stomata, as shown in Figure 3 [41]. Furthermore, signaling peptides CLAVATA3/ESR-RELATED (CLE) family genes *CLE9/10* regulate stomatal lineage development perceived by receptor kinase HAESA-LIKE 1 (HSL1) in a different pathway [42]. HSL1 recruits SERKs as co-receptors in the presence of CLE9/10 for different signaling modes during stomatal development, as shown in Figure 3 [42].

## 4. Feedback Regulation of SPCH, MUTE, and FAMA

SPCH, MUTE, and FAMA form a heterodimer with SCREAM 1 (SCRM1), SCRM2, and bHLH proteins [36,43,44]. INDUCER OF CBP EXPRESSION 1(ICE1)/SCRM2, as a scaffolding partner, facilitates MPK3/6-mediated phosphorylation of SPCH for its activity inhibition and consequent degradation [36,43,45]. MPK3/6-regulates phosphorylation and subsequent degradation of ICE1/SCRM2 during a process that plays a crucial role in stomatal cell fate specification [43]. Furthermore, *FOUR LIPS* (*FLP*) and *AtMyb88* constrain GMC division, and an outnumbered GCs phenotype was observed in *flp myb88*-defective mutants [46]. SPCH/SCRM, which is suppressed by ER/SERK/TMM/EPF-induced phosphorylation of MPK cascade, induces expression of *EPF2* and *TMM* in a negative feedback mechanism [12,33]. MUTE induces ERLI and suppresses EPF2, whereas ERL1, upon perceiving EPF1, regulates meristemoid transition into the GMC stage [20,26,32].

Additionally, cell cycle-related genes such as cyclins A and D (CYCA and CYCD, respectively) in association with CYCLIN-DEPENDENT KINASE A 1;1 and D1;1 (CDKA1;1 and CDKB1;1) play a key role in stomatal development [47,48]. CYCD4 participates in hypocotyl stomatal lineage divisions and CDKB1;1- and CDKA1;1-dominant negative forms, and CYCA higher-order mutants inhibit the division of GMCs [49,50,51,52]. MUTE upregulates cell cycle-related genets (CYCs and CDKs) by binding to their promoters to regulate the symmetric cell division of GMCs. FAMA binds to *CDKB1;1* promoter regions, and FLP suppresses the expression of *CDKB1;1* and *CDKA1;1.* As well, FAMA and FLP negatively regulate GMC symmetric division by repressing CDKs and CYCs [51,53,54]. Taken together, MUTE locks in the cells in the differentiation program, promotes regulators of the symmetric division of GMCs, and upregulates FLP and FAMA, which suppress regulators of the cell cycle, to control single symmetric cell division, as shown in Figure 3 [55].

## 5. Cell Cycle Regulators Join bHLH Transcription Factors to Control Stomatal Development

The SPCH, MUTE, and FAMA bHLH transcription factors interact with ICE1/SCRM and SCRM2, and other bHLH transcription factors through helix-loop-helix domains to form heterodimers [44]. Stomatal lineage progression and cell division are regulated via global transcriptional changes by SPCH, MUTE, FAMA, SCRM/ICE1, SCRM2, and closely related FLP and MYB88 [56]. These transcription factors join cell-cycle core regulators (cyclin-dependent kinases, CDKs) to control stomatal cell multiplication and differentiation throughout the lineage and specific developmental stage [52,56,57]. Cyclin-CDK couple regulates G1/S and G2/M transition phases during stomatal development [50,51]. In *Arabidopsis thaliana,* CDKA;1 and CDKB1s are known to regulate asymmetric and symmetric cell division during stomatal development, respectively [50,53,58]. CDKA;1 binds with D-type cyclins to facilitate cell entry from G1 into S phase by phosphorylating Retinoblastoma Related 1 (RBR1), a G1-S phase transition inhibitor [58]. CDKA;1-CYCD complex-mediated inhibition of RBR1 results in the release of E2F/DF, cell cycle-related transcription factors, to facilitate the expression of genes required for S phase entry [59].

In contrast, CDKB1;1-CYCA2;3 complex synergistically mediates GMC division by promoting G2-M phase transition [47,52]. Consequently, *cdkb1;1 cdkb1;2* double- and *cyca2;234* triple-defective mutants exhibit the single undivided GCs phenotype, which was further increased in *cyca2;234 cdkb1;1* quadruple mutants [52,53]. Furthermore, cdka;1 and MUTE promoter driving dominant-negative mutants displayed few single undivided GCs, which indicates that CDKA;1-CYCD3 complex activity is partially required in GMC symmetric division [51,60]. Consistently, overexpression of *TMM* promoter-driven *CDKA;1* partially rescued the *cdkb1;1 cdkb1;2* single undivided GCs phenotype [60].

SPCH-initiated asymmetric MMC division results in unequal SPCH degradation by protein kinase signaling cascade and scaffold polarity proteins peripheral localization, BREAKING OF ASYMMETRY IN THE STOMATAL LINEAGE (BASL) and POLAR LOCALIZATION DURING ASYMMETRIC DIVISION AND REDISTRIBUTION (POLAR), result in a small meristemoid and large SLGC daughter cells that become a stoma and a pavement cell, respectively [61,62,63].

In *Arabidopsis*, CDKA;1 is critical in G1-S phase entry and maintenance of stem cells; its loss of function mutant displays few GCs with enormously enlarged epidermal cells [58,60]. The depletion of RBR1 rescued the *cdka1* phenotype, and the RBR1 knockout displayed improperly divided meristemoids and stomatal lineage cells. These indicate that CDKA;1 and RBR1 act antagonistically during stomatal development [59,60]. Furthermore, CDKA;1 also phosphorylates SPCH on serine 186 residue, and its substitution with phosphomimetic residue resulted in overactive SPCH-mediated clustered stomata [64]. Hence, CDKA;1 acts in an antagonistic manner to a MAPK cascade, which inhibits stomatal development upon receiving extrinsic peptide signals [32,36,65]. Intriguingly, MAPKs also phosphorylates serine 186 of SPCH [36], indicating that cell cycle inhibitors (cell-cell signals and cell cycle machinery) regulate stomatal development at a SPCH posttranslational modification level. CDKA;1-phosphorylates RBR1, which suppresses SPCH and other stomatal lineage-related genes [17,58,60,64].

**Figure 4:**
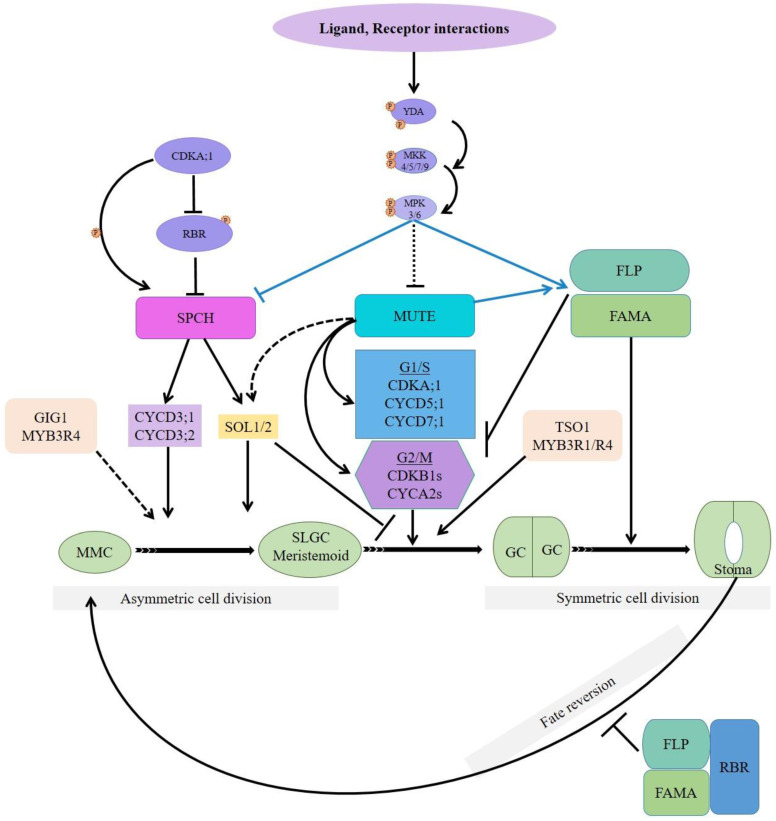
Molecular intersections, role, and mechanism of cell cycle regulators during stomatal formation. Sarcastically selected protodermal cells divide asymmetrically into large SLGC and small meristemoid compartments. Meristemoids, after several amplifying divisions, become a GMC, which divides into a pair of GCs with an opening in between them to form stomata. SPCH, MUTE, FAMA, and FLP/MYB88 regulate all these steps. CDKA;1 can phosphorylate both RBR1 and SPCH; CYCD3 cyclins are the direct targets of SPCH; SPCH expresses SOL1/2, which participates in meristemoid-to-GMC and subsequent symmetric cell division. GIG1 and MYB3R4 synergistically specify cell fate during stomatal lineage development. MUTE controls cell cycle-related core genes and their transcriptional suppressors. TSO1 expression is independent of SPCH or MUTE, physically interacts with MYB3R1, and promotes GMC’s symmetric division. Fate reversion of GCs to MMC requires FAMA/FLP-RBR1 interaction. Black arrows and solid lines mean the place where the factors execute. Dashed lines represent a potential role. T-ended lines indicate suppression.

SPCH induction increases CYCD3;1 and CYCD3;2, which are abundantly expressed in meristemoids and play an important role in the initial stage of stomatal lineage [12,66]. CYCD3 triple loss of function decreased meristemoids, amplifying SLGCs asymmetric spacing divisions, significantly reducing the number of leaf epidermal cells [67,68]. On the other hand, overexpression of CYCD3 resulted in an abnormal increase in cell number and ectopic cell division, demonstrating CYCD3’s role in regulating cell number and division in developing leaves [67]. Molecular intersections, role, and mechanism of cell cycle regulators during stomatal formation have been modeled in Figure 4.

## 6. Fate Transition and Cell Divisions in Stomatal Lineage

GIGAS CELL1 (GIG1), which negatively regulates anaphase-promoting complex/cyclosome (APC/C), is necessary for cell fate transition and subsequent mitotic cell division. GIG1 loss of function mutants displayed giant GIGAS cells with a mixed-cell fate [69]. GIGAS cells are jigsaw-puzzle-piece-shaped pavement cells but express SPCH, EPF2, TMM, E1728, and KAT1, markers of stomatal lineage and GCs. Interestingly, *spch*-defective mutants did not form GIGAS or GCs, which confirms their stomatal lineage origin. Originally, *gig1*-1 and *gig1*-2 allelic recessive mutants were obtained from a *myb3r4* mutant enhancer screen in a forward genetic screen [56,69]. *MYB3R4* transcription factor positive regulators are required for G2-M phase transition and mitotic cell division [69]. SPCH regulates the meristemoid phase of stomatal lineage by controlling the expression of thousands of genes, including its own negative regulators such as BASL, EPF2, TMM, SOL1, and SOL2 [12,33,70].

Key stomatal lineage regulator, SPCH, induced SOL1/2 mediate cell fate transition factors and post-SPCH identities [70]. SOL1/2 vanish just before asymmetric and symmetric cell division. Ectopic expression of SOL2-suppressed GMC division resulted in single undivided GCs. Consistently, *sol1 sol2* double defective mutants exhibit an increased number of small cells and paired stomata in the lineage’s early and late phase, respectively. Furthermore, inappropriately persistent MUTE expression after cell division in the *sol1 sol2* mutant demonstrates that sole MUTE expression in the absence of SOL1/2 is not enough for GMC fate determination [70]. Interestingly, TSO1, a homology of SOL1/2, expresses in the stomatal lineage with the opposite to SOLs activity; simultaneously, it is not a target of either SPCH or MUTE [12,55]. Knockout mutants of TSO1 in the *sol1 sol2* background rescues the *sol1 sol2* phenotype and decreases the number of small cells and paired GCs [70]. In conclusion, GIG1 and MYB3R4 synergistically specify cell fate during stomatal lineage development, SPCH induced SOL1/2 mediates fate transition, and TSO1 interacts with MYB3R1 and promotes GMC’s symmetric division (Figure 4).

## 7. Highly Conserved SnRK1 Positively Regulates SPCH-Mediated Stomatal Development

**Figure 5:**
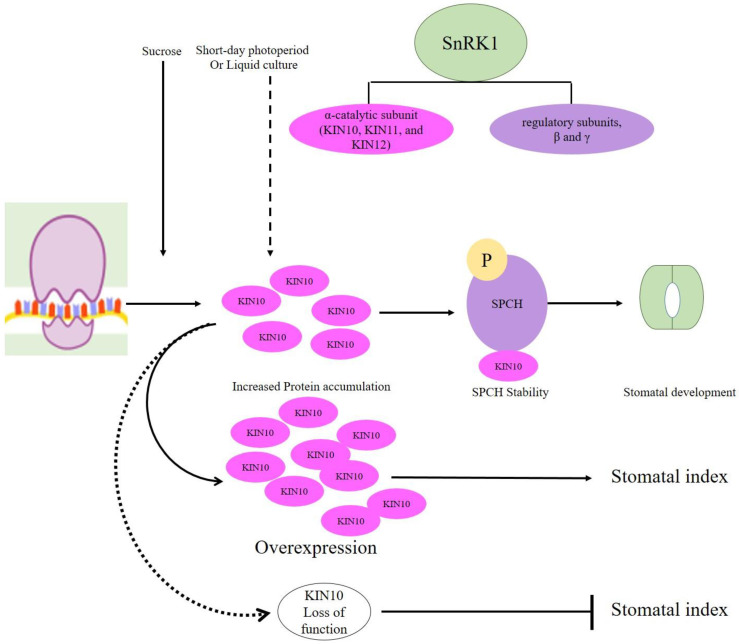
Sucrose non-fermenting-1 (SNF1)-related kinase 1 (SnRK1), especially subunit KIN10, promoted phosphorylation and stabilization of SPCH-mediates stomatal development under short photoperiod or liquid cultures (mild energy starvation of plants). Sucrose induces the accumulation of KIN10 protein by increasing its translation. Overexpression of KIN10 increases the stomatal index, whereas the loss of function of KIN10-decreases the stomatal index.

Energy hemostasis during stomatal development is controlled by sucrose non-fermenting-1 (SNF1)-related kinase 1 (SnRK1), which is a central energy sensor kinase in plants [71]. In *Arabidopsis*, SnRK1 functions as heterotrimeric complexes composed of one α-catalytic subunit (KIN10, KIN11, and KIN12) and two regulatory subunits, β and γ [72,73]. Sucrose-induced KIN10 expresses in all epidermal cells but exhibits cell-specific subcellular localization, accumulation in liquid culture conditions [73]. Furthermore, Han et al. (2020) report that stomatal lineage cells are highly enriched with nuclear-localized KIN10 that phosphorylates SPCH to increase stability and subsequent stomatal development under stress conditions. Overexpression of KIN10 increased, whereas KIN10 and KIN11 loss of function reduced stomatal index under short-day light or in liquid medium with 1% sucrose. However, *kin10* mutants displayed increased stomatal index on the solid medium (without sucrose) and low light conditions. Moreover, SPCH stability was greatly reduced by a mutation in KIN10 phosphorylation sites [73]. To sum up, SnRK1 α-catalytic subunit (KIN10) stabilizes SPCH, positively regulating stomatal development (Figure 5).

## 8. Protein Phosphatase 2A-Mediates SPCH Stability by Dephosphorylating It

Protein Phosphatase 2A (PP2A) heterotrimeric complexes comprise scaffolding subunit A, regulatory subunit B, and catalytic subunit C, with multiple isoforms for each subunit and differentially assembled complexes in *Arabidopsis* [74,75]. These complexes, differentially assembled from various isoforms of these A, B, and C subunits, regulate development, growth, metabolism, and stress responses in plants [75,76]. PP2A controls blue light-mediated stomatal movement, and in association with SnRK2, it regulates ABA-triggered stomatal closure [77,78]. PP2A function is required for the prophase band formation in *Arabidopsis* and the orientation of cell division in maize during stomatal development [79,80]. Recently, the role of PP2A-mediated positive regulation SPCH stability by direct interaction between SPCH and A subunit of PP2A to promote stomatal development has been reported [81].

**Figure 6:**
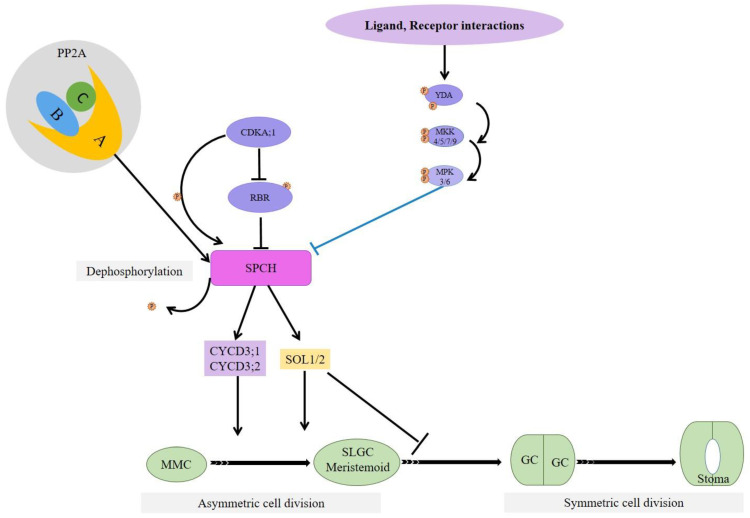
PP2A’s A subunit, which directly interacts with SPCH, in association with B and C subunits and SPCH dephosphorylates SPCH at a specific site. PP2A-induced dephosphorylation increases SPCH accumulation and stability.

Brassinosteroid (BR) promotes SPCH function by suppressing glycogen synthase kinase 3 (GSK3)/BRASSINOSTEROID insensitive 2 (BIN2), which are phosphorylating SPCH for subsequent degradation [36,82]. On the other hand, CYCLIN-DEPENDENT KINASE A;1 (CDKA;1)-mediated SPCH phosphorylation positively regulates SPCH function [64]. Bian et al. (2020) reported that PP2A increases SPCH accumulation and stabilization by direct interaction between A subunit and SPCH. PP2A dephosphorylates SPCH at a specific site. However, overexpression of PP2A-A did not exhibit a visible phenotype, which indicates that the other two subunits are also required for SPCH stabilization and subsequent consequences, as shown in Figure 6 [81].

## 9. IDD16 Represses SPCH-Induced Stomatal Initiation

The function of a C2H2 zinc finger transcription factor of the INDETERMINATE DOMAIN (IDD) family, encoded by AT1G25250, plays an essential role in organ-morphogenesis and gravitropic responses [83,84]. In *Arabidopsis*, C2H2 transcription factor IDD16 plays a critical regulatory role in stomatal development. IDD16 by trans-repression of SPCH negatively regulates stomatal initiation. In a dose-dependent manner, the overexpression of IDD16 reduced abaxial stomatal density in *Arabidopsis*. The initiation of stomatal lineage was significantly inhibited in IDD16 overexpression plants (IDD16-OE). Consistently, IDD16-OE seedlings displayed a severe reduction in SPCH levels. Conversely, the IDD16-RNAi transgenic line exhibited increased stomatal density, demonstrating that IDD16 is an intrinsic stomatal development regulator. Furthermore, ChIP analysis results showed that IDD16 could bind the promoter of SPCH [85]. With respect to the other eight down-regulated genes (MUTE, FAMA, TMM, SDD1, EPF1, EPF2, BASAL, and POLAR), which are expressed explicitly in MMC, meristemoid cells, GMCs, and GCs are attributed to reduced stomatal precursor cells development in IDD16-OE plants [85].

In some of the IDD16 overexpression transgenic lines, stomata could not form on the abaxial epidermis, whereas the adaxial side displayed a considerable number of stomata for survival and life cycle completion. Mutations in many of the stomatal development regulators such as SPCH, TMM, and SDD1 display similar changes in abaxial and adaxial epidermises [6,85]. The *tmm* and *sdd1* mutants displayed stomata in clusters and increased stomatal density and different adaxial-abaxial ratios [86]. A complex network of regulatory genes regulates the maintenance and acquisition of adaxial-abaxial leaf polarity, resulting in the asymmetric distribution of different cell types in mature leaves [87,88]. In *Arabidopsis*, pavement cell shape and pattern and stomatal density and pattern are different between both epidermises, which may be regulated by IDD16 [85].

## 10. Conclusions

We can conclude that SPCH promotes EPF2, the negative stomatal regulator, and TMM component stomatal complexes inhibit stomatal development in a negative feedback mechanism. In contrast, MUTE inhibits EPF2 and induces ER-family to promote SPCH-mediated stomatal lineage and development. Moreover, the PP2A A subunit, in association with its B and C subunits, increases SPCH accumulation, stability, and subsequent SPCH-induced stomatal lineage regulations. Similarly, SNRKs also positively regulate stomatal development by positively regulating SPCH, while IDD16 negatively regulates stomatal lineage initiation by suppressing SPCH.

## Data Availability

“Not applicable” for studies not involving humans.
